# G Protein–Coupled Estrogen Receptor GPER: Molecular Pharmacology and Therapeutic Applications

**DOI:** 10.1146/annurev-pharmtox-031122-121944

**Published:** 2023-01-20

**Authors:** Jeffrey B. Arterburn, Eric R. Prossnitz

**Affiliations:** 1 Department of Chemistry and Biochemistry, New Mexico State University, Las Cruces, New Mexico, USA; 2 University of New Mexico Comprehensive Cancer Center, University of New Mexico Health Sciences Center, Albuquerque, New Mexico, USA;; 3 Center of Biomedical Research Excellence in Autophagy, Inflammation and Metabolism, and Division of Molecular Medicine, Department of Internal Medicine, University of New Mexico Health Sciences Center, Albuquerque, New Mexico, USA

**Keywords:** cancer, cardiovascular, endocrine, estrogen, immunity, metabolism

## Abstract

The actions of estrogens and related estrogenic molecules are complex and multifaceted in both sexes. A wide array of natural, synthetic, and therapeutic molecules target pathways that produce and respond to estrogens. Multiple receptors promulgate these responses, including the classical estrogen receptors of the nuclear hormone receptor family (estrogen receptors α and β), which function largely as ligand-activated transcription factors, and the 7-transmembrane G protein–coupled estrogen receptor, GPER, which activates a diverse array of signaling pathways. The pharmacology and functional roles of GPER in physiology and disease reveal important roles in responses to both natural and synthetic estrogenic compounds in numerous physiological systems. These functions have implications in the treatment of myriad disease states, including cancer, cardiovascular diseases, and metabolic disorders. This review focuses on the complex pharmacology of GPER and summarizes major physiological functions of GPER and the therapeutic implications and ongoing applications of GPER-targeted compounds.

## INTRODUCTION

Estrogens elicit a multitude of effects throughout the body in virtually every organ, tissue, and physiological system. Although predominantly recognized as the female sex hormone, regulating sexual development at puberty, the menstrual cycle and pregnancy during the reproductive years, and, through the cessation of its synthesis, menopause, estrogen also has important and diverse roles in cardiovascular, metabolic, and neurologic functions as well as in many other systems. As a result of its critical functions in reproductive tissues (predominantly the uterus but also the breast), estrogen and its derivatives are employed in contraceptives, hormone replacement therapies for menopause, and the treatment of hormone-responsive (i.e., ER-positive) breast cancer. The diverse roles of estrogen are perhaps best exemplified by symptomatic and physiological changes experienced by women following menopause that include loss of periods, vaginal dryness, urinary incontinence, loss of breast fullness, hot flashes/chills/night sweats, sleep difficulties, mood changes, weight gain/slowed metabolism, thinning hair, and dry skin (1). However, additional roles for estrogen are revealed by the increased risk following menopause, and the decreased risk following hormone replacement, of a multitude of diseases, including cardiovascular diseases (e.g., coronary artery disease, hypertension, stroke), osteoporosis, obesity and dyslipidemia, diabetes, and neurological changes (e.g., depression and dementia) (2, 3). Estrogen also plays a critical role in about 80% of breast cancers, in which tumor growth is stimulated by and often dependent upon estrogen. This estrogen dependence has led to diverse therapeutic approaches to treat breast cancer that include inhibiting the production of estrogen via the enzyme aromatase and targeting one of its receptors (ERα) through either inhibition or degradation (4, 5).

The pharmacology surrounding estrogen receptors is diverse and complex (6, 7). In addition to the multiple forms of estrogen produced in the human body [predominantly estrone (E1), 17β-estradiol (E2), estriol (E3), and estetrol (E4) (8)], natural and manmade xenoestrogens elicit estrogenic activity (9, 10). The definition of stimulating effects in the uterus (imbibition and proliferation as standardized end points), as E2 does, is practical but neglects broader effects, with little consideration of actions in other tissues. Natural plant- or fungus-derived and manmade xenoestrogens, also referred to as environmental estrogens or endocrine-disrupting compounds, are ubiquitous in the environment and diet and have impacts on biology and human health (9, 11). Drugs targeting estrogen levels/synthesis and receptor activity play a role in the treatment of many conditions and diseases (12, 13), but particularly cancer (5, 14). Thus, understanding the mechanisms of action with respect to the multiple estrogen receptors is of critical importance. In this review, we describe the pharmacology and therapeutic implications of these diverse compounds with particular reference to their actions via the 7-transmembrane G protein–coupled estrogen receptor (GPER).

## ESTROGEN RECEPTORS: ERα/β AND GPER

Two distinct receptor families mediate estrogen’s diverse transcriptional (i.e., genomic) and rapid signaling (i.e., nongenomic) activities (6, 7). Although early experimentation identified estrogen-induced rapid signaling [e.g., cyclic adenosine monophosphate (cAMP) production and Ca^2+^ uptake], the transcriptional activities of ER soon dominated the field. Continued reports of the rapid actions of estrogen and other steroids led to the hypothesis of membrane-associated forms of ER in the 1990s (15). In 1996, a receptor homologous to ER was cloned and functionally shown to be a second ER, leading to the current nomenclature of ERα and ERβ (16, 17), while concurrently an orphan 7-transmembrane-spanning G protein–coupled receptor (GPCR) was cloned and termed GPR30 (18). In 2000, GPR30 was shown to mediate rapid activation of extracellular signal-regulated kinase (ERK) in response to estrogen, providing the first evidence for its actions as a functional estrogen receptor (19). This discovery was followed by the demonstration of specific estrogen binding, employing both tritiated (20) and fluorescent derivatives (21) in 2005, leading to the official designation of GPR30 as GPER by the International Union of Basic and Clinical Pharmacology in 2007 (22). Demonstration of its activity as a classical GPCR was provided by the effect of guanosine-5′-triphosphate (GTP) (specifically GTPγS, via activation and dissociation of heterotrimeric G proteins) on reducing ligand binding through conversion of the receptor to a lower affinity state as well as by increased GTPγS binding in the presence of estrogen (20).

As a GPCR, GPER’s primary site of subcellular localization, the endoplasmic reticulum and Golgi apparatus (21), is unusual although not unique (23). In some cells, detectable GPER is found at the plasma membrane, although even in such cells, most is present in intracellular membranes at steady state (23). As estrogens are cell permeable (24, 25) and activate ERs intracellularly, and as most ERα is localized within the nucleus at steady state (26), studies with permeable and non-permeable estrogen derivatives suggest that GPER signals predominantly from an intracellular location(s) (27). Receptor trafficking studies suggest that GPER expressed at the cell surface is constitutively internalized in a ligand-independent manner, consistent with the majority of the receptor being observed intracellularly at steady state (23).

Signaling initiated by GPER occurs through a multitude of pathways. Coupling occurs through multiple heterotrimeric G proteins, primarily Gαs (28) and Gαi (21), as well as Gβγ-mediated signaling (19). In addition, much if not all signaling initiated by GPER activation involves transactivation of the epidermal growth factor receptor (EGFR) (19), a pathway described for many GPCRs (29). This pathway involves Gβγ-mediated activation of Src, leading to α5β1 recruitment and matrix metalloproteinase (MMP)-mediated release of heparan-binding EGF-like growth factor, which then transactivates EGFR, with ensuing activation of multiple additional pathways such as ERK and PI3K/Akt (29). Whereas ERK activation leads to proliferative signaling and Elk-1-mediated transcriptional regulation (30), Akt activation leads to phosphorylation of both eNOS (31), leading to NO production, and Foxo3 (32), leading to prosurvival signals. GPER activation also leads to adenylyl cyclase activation, producing cAMP, which in turn activates protein kinase A (PKA), and transcriptional events via cAMP response element-binding protein (CREB) (33, 34). Thus, although signaling via GPER is widely considered to mediate rapid nongenomic signaling, the downstream events of these early signaling events include extensive genomic regulation, much as ER-mediated signaling involves rapid events in addition to its classical transcriptional regulation.

## GPER LIGANDS AND PHARMACOLOGY

Promiscuous ligand binding with many different structural classes and diverse pharmacology are characteristics of both classical (nuclear) ERα/β and GPER. The most potent estrogenic hormone, E2, is a lipophilic phenol, and compounds featuring this functionality are frequently cross-reactive ligands. The identification and characterization of pharmacologically active GPER ligands were extensively reviewed in 2015 (7), and recent developments and discoveries with the potential of impacting human health and clinical applications are the focus of this review. The scope of compounds with recognized biological effects through GPER continues to grow and includes US Food and Drug Administration (FDA)-approved drugs and chemicals ingested in food, nutritional supplements, and other environmental exposures. It is important to recognize that ligands with widely disparate GPER-binding affinities can regulate diverse nongenomic signaling pathways that ultimately impact genomic outcomes. This scenario presents challenges for interpreting gene expression and toxicological effects that may be observed at doses that are significantly lower than measured affinities or activities would predict, in alignment with observations that endocrine disruptors frequently exhibit nonmonotonic dose-response relationships (35).

### GPER Pharmacology with Natural Steroids and Derivatives

**Figure 1:**
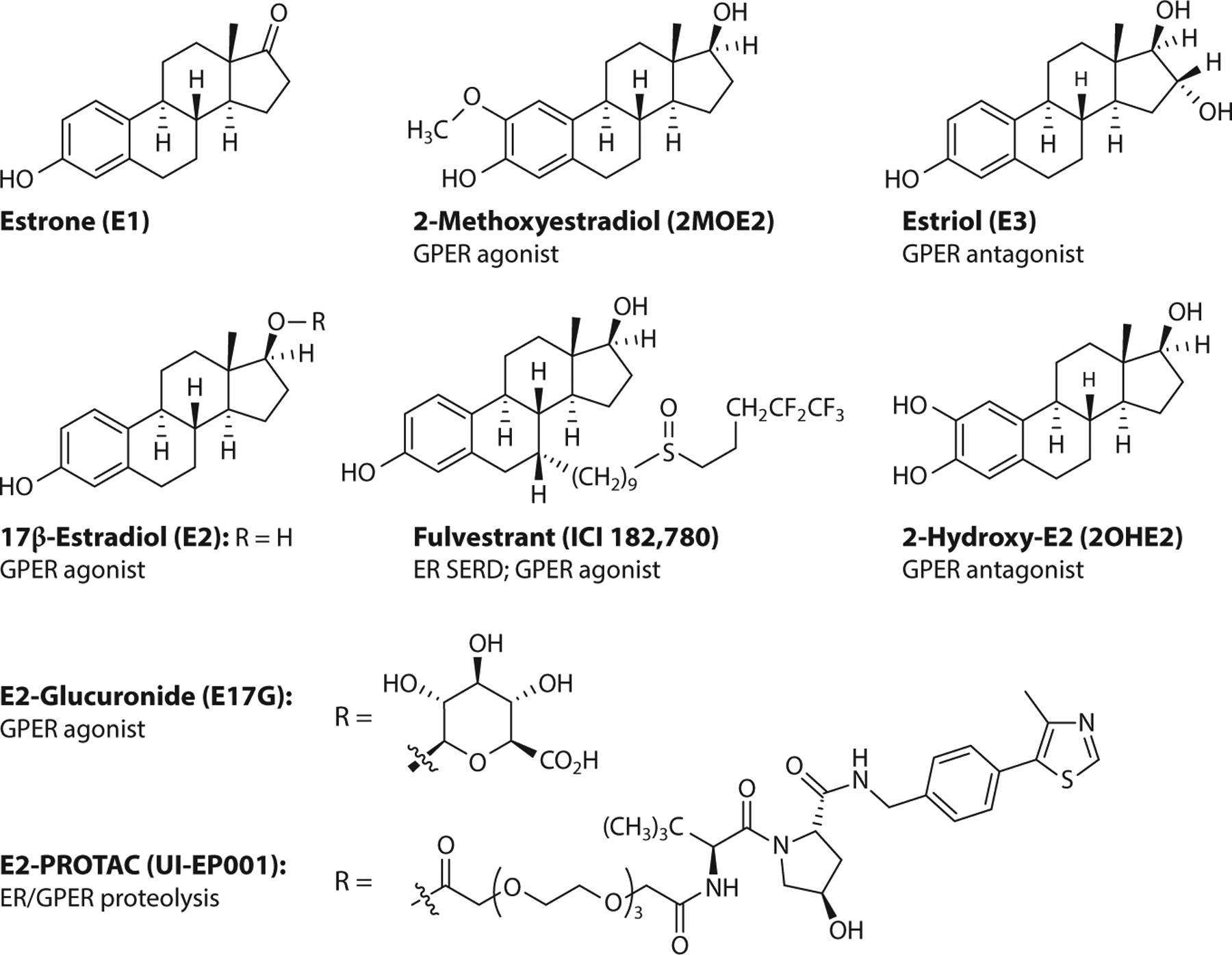
Steroidal ligands of GPER. Abbreviations: ER, estrogen receptor; GPER, G protein–coupled estrogen receptor; PROTAC, proteolysis-targeting chimera; SERD, selective estrogen receptor downregulator/degrader.

Competitive binding assays with radiolabeled or fluorescent probes revealed that E2 has the highest GPER binding affinity (3–6 nM) and greater than 1,000-fold selectivity compared to other steroid hormones such as progesterone, testosterone, and cortisol (20). Whether and how aldosterone may act in concert with or through GPER remain complex and controversial questions (36, 37), particularly given the demonstrated lack of binding (38). The physiologically relevant estrogen E1 has much lower affinity for GPER (>10 μM) (20). The 16α-hydroxy analog E3 (20) and the catechol metabolite 2-hydroxy-17β-estradiol (39) have relatively low GPER binding affinities (>1 μM and 0.1–1 μM) but function as weak antagonists (Figure 1). In contrast, the more lipophilic metabolite 2-methoxyestradiol exhibits relatively high affinity (10 nM) and functions as an agonist (40–44). The oxysterol 27-hydroxycholesterol has recently been demonstrated to bind GPER (with an affinity of approximately 1 μM) and function as an agonist in ER-negative breast cancer cells (45). The 17β-d-glucuronide metabolite of E2 has low GPER binding affinity (>50 μM) and reported agonist activity (46), but interpretations of results from these types of conjugates are complicated by the susceptibility to chemical or enzymatic hydrolysis releasing E2. Similar cautions are appropriate using dehydroepiandrosterone (DHEA) in cells and particularly in vivo studies where biosynthesis to produce E2 can occur (47). The synthetic estrogen derivative fulvestrant [a selective estrogen receptor downregulator/degrader (SERD)] functions as a pure ERα antagonist but also induces ERα degradation due to conformational changes induced by the extended 7α appendage in the ligand-bound structure. This drug is FDA approved for advanced ER-positive breast cancer but also acts as a GPER agonist (19), illustrating the need for including GPER when profiling receptor selectivity to develop more selective drugs with fewer potential off-target effects. ERα and GPER binding, functional responses, and ligand localization of E2 conjugates with fluorescent dyes or chelates have been employed to quantitate, characterize, and visualize ligand binding and function at the subcellular/cellular (21, 27) and organismal levels (48), respectively. Proteolysis-targeting chimeras (PROTACs), based on small molecules linked to an E3 ubiquitin ligase ligand that degrades the target, are under evaluation as a strategy for the development of novel cancer therapeutics (49), with nuclear receptors offering an important target (50). Estrogen chimerae (E2-PROTACs), first described in 2005 (51), have recently been reported to bind both GPER and ER with relatively high affinity (~ 30 nM and 10–20 nM, respectively), resulting in the degradation of ERα/β as well as GPER in MCF7 and SKBR3 cell lines without affecting progesterone receptor levels (52). PROTACs provide an alternative approach for targeting plasma membrane and intracellular estrogen receptors that could enable receptor-selective degradation based on selective receptor ligands.

### Xenoestrogens as GPER Ligands

There is growing recognition of the role of GPER in endocrine disruption through exposure to natural and synthetic xenoestrogens originating from dietary intake, health and nutritional supplements, and environmental exposures to agrochemicals and industrial compounds, including polymers and their degradation products. The number of recognized xenoestrogens is staggering, and while previous studies have primarily focused on ERα/β, many of these compounds activate GPER, with possible consequences on neurogenic processes (53), and cancers of the breast (54), prostate (55) and digestive system (56). The breadth of possible GPER ligands was made apparent by a study that virtually screened a database of 30,926 natural products and identified 500 compounds, representing diverse structural classes that included flavonoids, isoflavonoids, chalcones, coumestans, stilbenes, lignans, ginsenosides, and tetrahydrofurandiols (57). The presence of a phenol and hydrophobic scaffold is a characteristic feature of many xenoestrogenic compounds.

**Figure 2:**
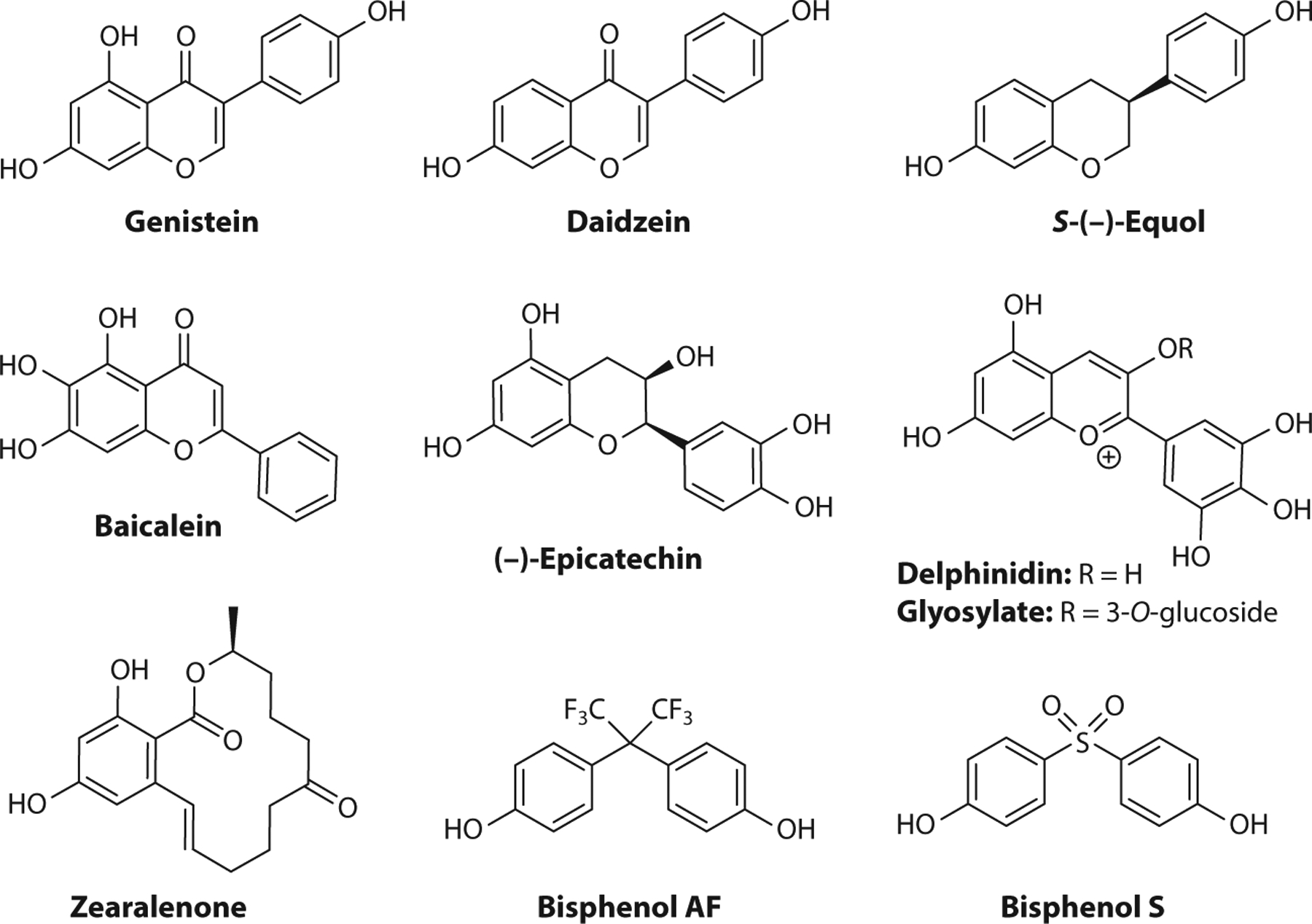
Xenoestrogen ligands of G protein–coupled estrogen receptor (GPER).

The isoflavones genistein and daidzein are phytoestrogens that are widely consumed directly from soy products and often taken as medicinal supplements with the intent of easing menopausal symptoms, improving metabolism, reducing cardiovascular disease, or preventing certain hormone-related cancers (Figure 2). These compounds possess a phenol group at the 3-position of the 4H-chromen-4-one core and bind to GPER with high affinity. The crystalline sodium salt dihydrate of genistein, designated AXP107–11, sensitized gemcitabine chemotherapy in pancreatic ductal adenocarcinoma patient-derived xenografts synergistically through activation of GPER and mitogen-activated protein kinase (MAPK) signaling (58). GPER has been implicated in studies demonstrating that genistein attenuates inflammation in a model of Parkinson’s disease, inhibiting microglial activation and protecting dopaminergic neurons (59); protects against oxidative stress in hepatocytes (60); and improves glucose tolerance and white adipose tissue thermogenesis (61). Daidzein is converted to *S*-(−)-equol by mammalian gut bacteria, and individual metabolic variations result in widely ranging exposures. *S*-equol targeted GPER to promote glucose-induced insulin secretion from pancreatic β cells and prevented glucagon-like peptide-1 secretion from enteroendocrine L cells (62); activated GPER signaling, with effects on vascular smooth muscle cells (63); inhibited nitric oxide production and reduced expression of inducible NO synthase in lipopolysaccharide-stimulated astrocytes (64); and induced cell proliferation and migration in astrocytes that were attenuated by the GPER antagonist G15 but not by the SERD/GPER agonist fulvestrant (65). Genistein, daidzein and *S*-(−)-equol increased glial cell migration through activation of GPER signaling, and molecular docking studies suggest that these three compounds may bind to GPER at the same position as E2 (65).

The isomeric flavones feature 2-aryl substituents on the 4H-chromen-4-one core, and these compounds are further metabolized to various phytoestrogens. Baicalein occurs in an herb used in traditional Chinese medicine and, with a simple 2-phenyl substituent, functioned as a GPER antagonist to reduce E2-induced migration, adhesion, and invasion in breast cancer cells (66) and suppressed E2-induced cell invasion and matrix metalloproteinase-9 expression and activation (67).

Polyphenolic catechins such as (−)-epicatechin occur in green tea, cacao, and pome fruits and have attracted significant attention regarding their potential health benefits. (−)-Epicatechin activated GPER signaling pathways for vasodilation similarly to G-1 (68) and stimulated mitochondrial biogenesis in mouse skeletal muscle (69). Synthetic propargylic ether derivatives of (−)-epicatechin retained activity in the eNOS/NO pathway and, when immobilized, functioned as an affinity column to pull down GPER from protein extracts of endothelial cells, further validating (−)-epicatechin as a GPER ligand (70).

Anthocyanins are a diverse class of highly colored flavonoids found in fruits and red wine that have been of interest for their nutraceutical value and potential benefits for vascular disease. The aglycone delphinidin and the glycosylate delphinidin 3-glucoside were equipotent in eliciting rapid NO-mediated vasodilator responses in male rats, and this response was mimicked by tissue perfusion with either G-1 or E2 and significantly reduced by treatment with G36, which implicated GPER in this pathway (71).

Zearalenone is a phenolic macrolactone produced by mycotoxins in grains and cereals and is metabolized to the epimeric alcohols α- and β-zearalenol. With widespread occurrence, these compounds are consumed by animals and humans, raising concerns of estrogenic effects on the reproductive system, other toxicities, and potential roles in the development of hormone-dependent cancers. Zearalenone is a GPER agonist, and exposure in pig pituitary cells and glands caused increased expression of GPER messenger RNA, but not ERα/β, along with activation of GPER/PKC/p38 pathways to upregulate the microRNA miR-7, which targets the *FOS* gene, leading to inhibition of follicle stimulating hormone synthesis and secretion and resulting in reproductive defects (72, 73). In colon cancer cell lines, which are not typically considered hormone sensitive, zearalenone promoted anchorage-independent cell growth and cell cycle progression, which was suppressed by G15, via MAPK and Hippo pathway effector YAP1, providing a mechanism for promoting colon cancer growth (74).

Multiple structural classes of synthetic endocrine-disrupting chemicals are ligands for GPER, and a pharmacological screening approach was developed to distinguish GPER agonist/antagonist activities. This method used live-cell imaging to monitor changes in the morphology of MR5C human fibroblast cells in response to G-1 and G15 to evaluate GPER activity (75). Bisphenols are produced industrially and incorporated into many consumer products on a massive scale worldwide and are one of the most important classes of endocrine-disrupting compounds. These analogs generally exhibit higher relative binding affinities for GPER than ER and initiate extranuclear signaling pathways at low doses that challenge their classical designation as weak estrogens (76, 77). The fluorinated bisphenol analog BPAF had ninefold higher affinity for GPER than the parent compound, determined using a fluorescent competitive binding assay in GPER-expressing SKBR3 cells, and GPER-mediated nongenomic effects were observed at 10-nM concentrations (78). As manufacturers move to BPA-free alternatives with analogs such as the sulfone BPS, the concerns for estrogenic activity remain and warrant further study in the broad context of involved receptors and increased diligence for monitoring and risk assessment.

### Synthetic GPER-Targeted Compounds

**Figure 3:**
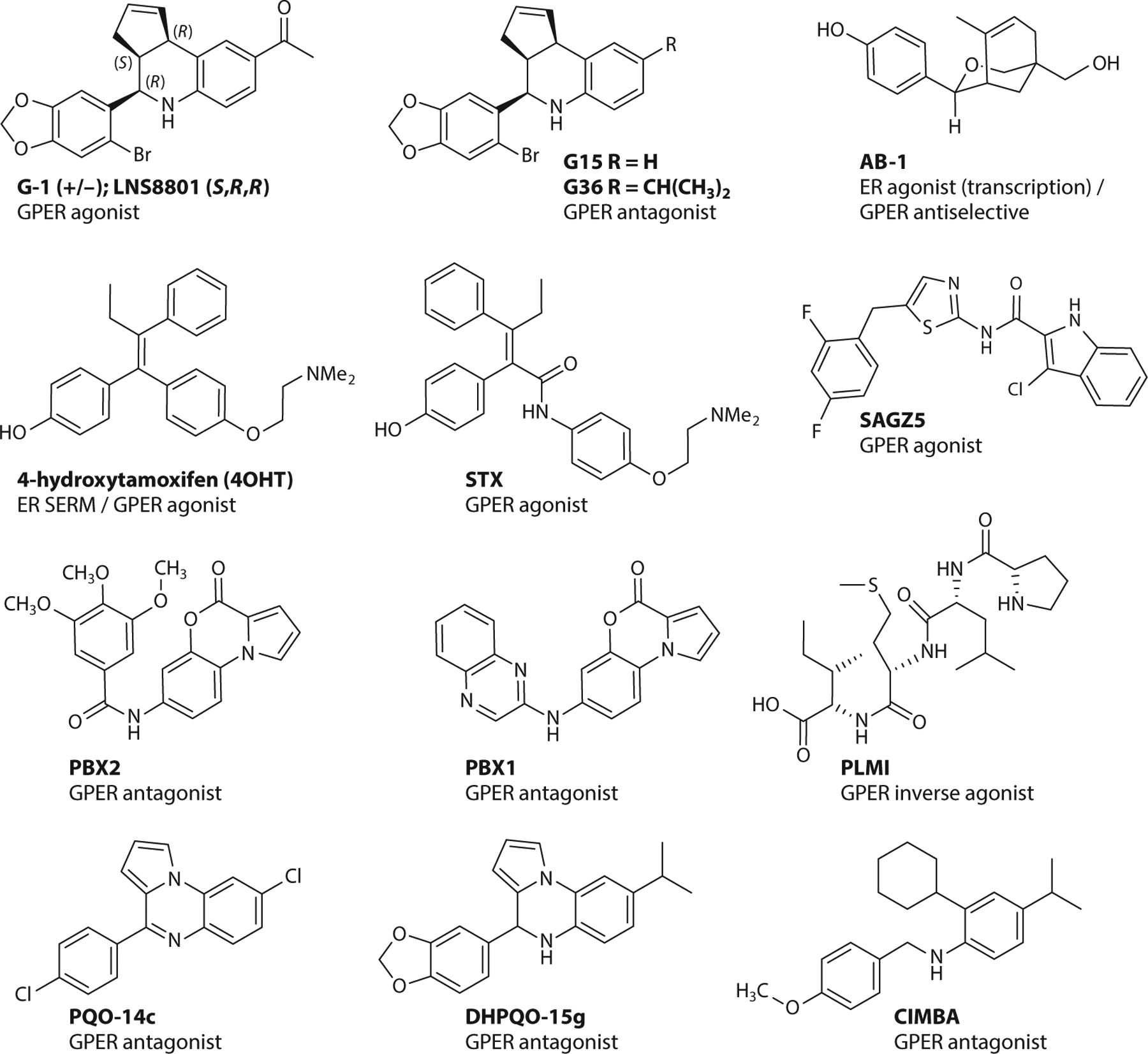
Synthetic heterocycles with selectivity for GPER. Abbreviations: ER, estrogen receptor; GPER, G protein–coupled estrogen receptor; SERM, selective estrogen receptor modulator.

The discovery that GPER could play a role in the activities of estrogenic compounds (19–21, 79) established the critical need for new pharmacological tools to distinguish between the activities of ERα/β and GPER. The challenges of obtaining atomic-resolution structures for membrane-bound GPCRs, and the absence of such a structure of GPER, have been significant impediments for structure-based approaches seeking to design and optimize GPER-targeted compounds. Combined virtual and biomolecular screening approaches resulted in the discovery of the first, and to date the most widely studied, GPER agonist, G-1 (80) (Figure 3). This strategy employed a ligand-based computational screen of a 10,000-member compound library for structural similarity with E2 to rank compounds for cell-based flow cytometry competitive binding assays, which employed a fluorescent synthetic E2-probe to distinguish compounds exhibiting selective GPER binding with respect to the nuclear receptor subtypes (80). Subsequent application of synthetic medicinal chemistry for structure-activity studies of the tetrahydro-3H-cyclopenta[*c*]quinoline scaffold resulted in the identification of the first GPER antagonist, G15 (81), and the improved analog, G36 (82). The activity, selectivity, and GPER dependence of G-1 have been demonstrated in a number of ER-negative cell lines, including SKBR3 (breast cancer) (19), Hec50 (endometrial cancer) (83), and MCF10A (normal breast epithelium) (84) cells, employing small interfering RNA knockdown approaches (32, 83, 84) as well as in multiple systems in GPER knockout (KO) mice (85). To date, the activities of these compounds, where examined, are absent in cells and mice lacking GPER (85), with the exception of reported effects on tubulin at high concentrations (3–50 μM) (86, 87). These validated GPER-selective G-series compounds are commercially available as racemic mixtures and have enabled the application of molecular biology approaches and a wide variety of in vitro and in vivo studies for characterizing new ligands and distinguishing GPER from ERα/β in multiple pathways in diverse cell, tissue, and organ types. The (*S*,*R*,*R*)-enantiomer of G-1 [1-((3a*S*,4*R*,9b*R*)-4-(6-bromobenzo[d][1,3]dioxol-5-yl)-3a,4,5,9b-tetrahydro-3H-cyclopenta[*c*]quinolin-8-yl)ethan-1-one] was obtained by chiral (high-performance liquid) chromatography and has advanced as the first GPER-targeted investigational new drug (IND), LNS8801 (88), to enter human clinical trials (https://clinicaltrials.gov/ct2/show/NCT04130516).

The approach used to identify the G-series compounds also led to the discovery of the oxabicyclic compound AB-1, which exhibits a unique and inverse selectivity profile compared to G-series compounds, not binding to or affecting the activity of GPER, while acting as an agonist of ERα/β classical genomic responses/transcription (and antagonizing rapid nonclassical signaling pathways mediated by ER) (89). This selectivity is particularly notable considering that many ER-targeted compounds also interact with GPER; for example, the selective estrogen receptor modulator (SERM) (4-hydroxy)tamoxifen (the active metabolite of tamoxifen employed in in vitro experiments) is a potent agonist of GPER (20, 21, 90). The structurally related diphenylacrylamide tamoxifen-raloxifene hybrid, STX, also activated GPER in mHippoE-18 hippocampal clonal cells (37, 91).

Considerable effort has focused on the development of computational homology models for GPER, which have enabled molecular docking studies, molecular dynamics simulations, and virtual screening approaches, as described in recent reviews (92–101). While in-depth coverage of this subject is beyond the scope of this review, and characterization of many of the compounds remains incomplete, it is instructive to survey select new ligands that have been identified and summarize accompanying insights with respect to binding and function.

The structural significance of the G-1 scaffold as a pharmacophore has been established through several synthetic programs generating derivatives that retain GPER binding. The cyclopentene group has been saturated and replaced with tetrahydrofuranyl and tetrahydropyranyl groups (98, 102, 103). The methylketone group has been replaced by carboxylate and carboxamide functional groups (97, 104), and biaryl derivatives of the 5-bromobenzo[1,3]dioxole group have been prepared by Suzuki–Miyaura cross-coupling (94). In another example, a fluorescent borondipyrromethene difluoride (BODIPY) dye conjugated to the 6-position of a 5-bromobenzo[1,3]dioxole group mimicked the structure of G-1 and exhibited competitive ligand binding to GPER with ^3^H-E2 and G15 in SKBR3 cells (105). The amide-linked indole-thiazole SAGZ5 was identified by virtual screening of a 3D pharmacophore model and found to be a GPER agonist that activated adenylate cyclase and subsequent cAMP formation in HL60 cells with EC_50_ values similar to those of G-1 (106). The docking model proposed that SAGZ5 binds in the same hydrophobic site as modeled for G-1.

Several additional GPER antagonists with some structural similarities to G15 and G36 have been identified. The pyrrolobenzoxazinone compounds PBX1 and PBX2 were identified as GPER ligands by competitive binding studies and at 10-μM concentrations inhibited SKBR3 cell proliferation and cell migration of cancer-associated fibroblasts induced by 100 nM E2 and G-1 (107). Additional structurally related compounds such as pyrrolo[1,2-a]quinoxaline and dihydopyrrolo[1,2-a]quinoxaline (PQO-14c and DHPQO-15g) were identified as GPER antagonists using a homology model based on the chemokine receptor CXCR4 and by virtually screening for compounds with binding modes similar to the G-series of compounds (108). These compounds induced cell death in GPER-expressing MCF7 and SKBR3 cells, with structural analogs exhibiting differential effects on the expression of p53 and p21. Structurally related analogs of this scaffold inhibited cell proliferation in TNBC cells, with increased activity of a dihydropyrrolo derivative observed (109).

A homology modeling approach was used to design the benzylic aniline CIMBA that inhibited G-1-induced calcium mobilization (110). The structure of CIMBA can be considered as an acyclic analog of G36, providing increased conformational flexibility and aqueous solubility compared with the quinoline scaffold. Intraperitoneal injection of CIMBA in an ovariectomized mouse model prevented E2-induced cholesterol gallstones in a dose-dependent manner. These results encourage further study of GPER antagonists for the development of new drugs for treating cholesterol gallstone disease in women.

A peptide corresponding to residues 295–311 from the hinge region/AF2 domain of ERα (termed ERα17p) induced apoptosis in breast cancer cells and promoted regression in an ERα-negative tumor xenograft model (111). This peptide was suggested to be an inverse agonist of GPER, decreasing phosphorylation of EGFR and ERK1/2, decreasing c-fos expression, and inducing the proteasome-dependent downregulation of GPER (112). This activity was replicated by the short synthetic tetrapeptide PLMI, which is based on the N terminus of the larger peptide, and while at first consideration these peptides appear strikingly different from the other heterocyclic scaffolds, molecular docking studies suggested a correlation in the predicted GPER binding sites of the heterocyclic antagonist PBX-1 compound (112).

## THERAPEUTIC OPPORTUNITIES FOR GPER-SELECTIVE LIGANDS

### Cancer

**Figure 4:**
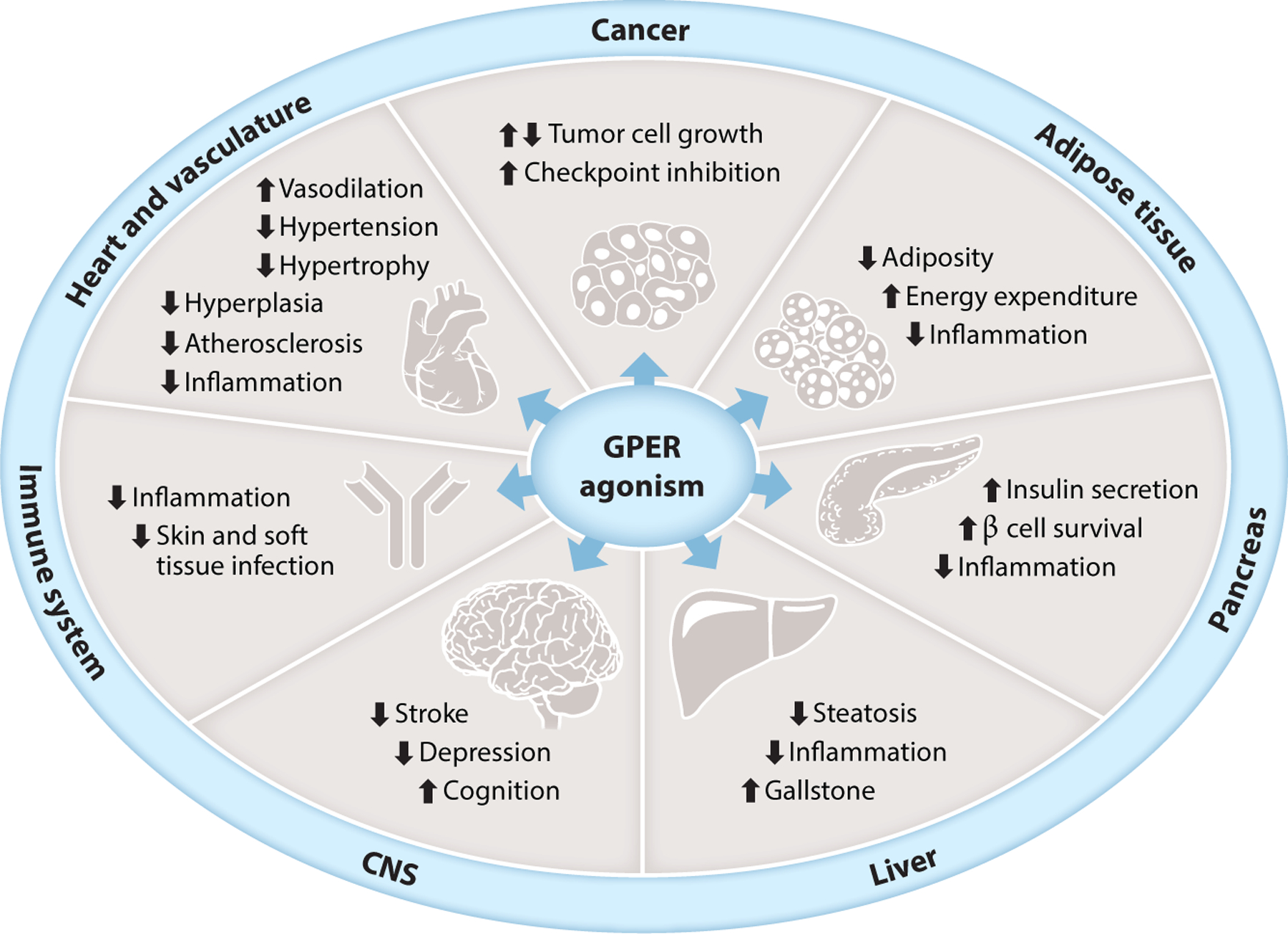
Examples of physiological effects resulting from selective GPER agonism. Abbreviations: CNS, central nervous system; GPER, G protein–coupled estrogen receptor.

GPER is expressed in a wide range of human cancers, suggesting possible roles for diagnosis, prognosis, or targeting its activity or expression as therapeutic interventions. GPER expression has been documented in human cancers (or cell lines) such as breast, endometrial, ovarian, prostate, pancreatic, thyroid, colon, lung, renal, and melanoma, among many others (reviewed in 113). In many cancer cell lines, including breast (84), endometrial (114), thyroid (115), and ovarian (116), G-1 promotes proliferation and associated signaling pathways (Figure 4). However, inhibition of proliferation has also been reported in breast (117), melanoma (118), prostate (119, 120), pancreatic (121), and other cancer cell lines. In a murine xenograft prostate cancer model encompassing both androgen-sensitive and castration-resistant cancer, G-1 inhibited cancer progression but only in castration-resistant disease (119, 120). Differences in mechanisms of cellular proliferation as well as G-1 concentrations used may account for these in vitro differences.

In humans, GPER expression correlates with poor outcome in breast (122–124), endometrial (125), and ovarian (126) cancers. GPER expression is increased in breast cancer metastases compared to matched primary tumors (127, 128) but, interestingly, only in women treated with tamoxifen (128). GPER expression also correlates with decreased tumor growth inhibition in primary ER-/GPER-positive breast tumors treated with tamoxifen compared to aromatase inhibition. This difference is absent in primary ER-positive breast tumors that do not express GPER (123, 124). The role of global GPER expression has been evaluated in the MMTV-PyMT murine model of spontaneous mammary tumorigenesis. Compared to wild-type mice, GPER KO mice yielded smaller tumors with reduced metastasis, which suggests that, in vivo, GPER has a protumorigenic function (129). Whether this finding is due to expression in the tumor cells or stromal cells (e.g., immune cells or fibroblasts) remains unknown.

As an agonist of GPER, (4-hydroxy)tamoxifen’s effects on breast cancer (cells) have been widely examined and are complex. Tamoxifen-resistant MCF7 cells proliferated in response to tamoxifen through a GPER-dependent pathway (127, 130), which was blocked by either GPER knockdown or G15 treatment (81, 127). Tamoxifen elicited cytoplasmic translocation of the proapoptotic transcription factor Foxo3, which may in turn contribute to resistance mechanisms (32, 90). Tamoxifen also induced breast cancer cell migration (131) and increased aromatase expression in tamoxifen-resistant cells (132) via GPER. In vivo, tamoxifen-resistant MCF7 xenografts regained sensitivity to tamoxifen upon treatment with G15 (127). G15 sensitized breast cancer cells to doxorubicin by inhibiting epithelial–mesenchymal transition (133). Finally, G-1 (as well as tamoxifen and fulvestrant) increased natural killer cell–mediated killing of both ER-negative and ER-positive breast cancer cells, suggesting yet another possible role for GPER in immune regulation (134).

In vivo effects of GPER agonists and antagonists are complicated by the widespread expression of GPER beyond tumor cells, including in tumor-associated immune and stromal cells (such as fibroblasts, adipocytes, and vascular cells). Anti-inflammatory effects of GPER and G-1 likely affect cancer initiation and early progress, as evidenced by accelerated inflammation-driven liver tumorigenesis in GPER-deficient mice (135). GPER expression in breast cancer–associated fibroblasts also suggests a role in cancer progression (136–138), where it promoted migration and invasion of cancer cells (139–141). Adipocytes in fat-rich tissues such as the breast and in the obese (142) also contribute to carcinogenesis of multiple cancers (143). Adipocytes express aromatase, yielding increased local estrogen levels as well as many adipokines and generally proinflammatory cytokines and hormones that can promote tumorigenesis. As G-1 reduces obesity and metabolic dysfunction (144), inflammation (113, 145), and chemotherapy-induced cardiotoxicity (146), it may reduce the incidence of or improve outcomes in breast and other cancers through diverse mechanisms.

GPER also plays important roles in many other cancer types. G-1 reduced liver tumorigenesis, in part through inhibiting inflammation and fibrosis (135). In contrast, in non-small-cell lung cancer, tumor burden increased with E2 or G-1 treatment and decreased with G15 treatment (147, 148). In melanoma cells, G-1 (as well as tamoxifen) inhibited proliferation in vitro (149), and when combined with anti-PD-1 antibody therapy, G-1 priming led to reduced tumor growth, substantially improving survival of melanoma-bearing mice (118). G-1 combination with immune checkpoint inhibition therapy also showed efficacy in pancreatic cancer models (121). These combined therapies led to immune memory, protecting against tumor rechallenge, suggesting broad effects in tumor and immune cells (118). These studies led to IND approval for G-1 in cancer and subsequent initiation of the first Phase I clinical trial of G-1 in 2019 (https://clinicaltrials.gov/ct2/show/NCT04130516).

### Cardiovascular System

Estrogens play important roles in the regulation of cardiovascular function, and their receptors therefore represent potential targets for therapeutic interventions in multiple cardiovascular diseases, including myocardial infarction (coronary heart disease), atherosclerosis, arterial and pulmonary arterial hypertension, and heart failure. The role(s) of estrogen is exemplified by the lower incidence of hypertension and coronary artery disease in premenopausal women compared to age-matched men and the substantial increase in both diseases following menopause (150, 151). Roles for GPER in the regulation of cardiovascular function and disease have been widely demonstrated using G-1 and include the regulation of blood pressure, angiogenesis, myocardial function, and inflammation (152).

G-1, like E2, induced vasorelaxation largely through nitric oxide production within multiple vessels (of rodent, porcine, and human origin) and acutely lowered blood pressure in mice, an effect that was absent in GPER KO mice (31, 71, 153–155). In salt-dependent hypertension with early diastolic dysfunction (heart failure with preserved ejection fraction), employing the mRen2.Lewis rat, chronic G-1 treatment improved myocardial relaxation in ovary-intact and ovariectomized females and reduced cardiac myocyte hypertrophy and wall thickness, in the absence of overt changes in blood pressure (156, 157). Similar therapeutic effects of G-1 occurred in aged rats (158) and in AngII-induced hypertensive rats (159). G-1 treatment (for 2 weeks at 14 months of age) also reversed hypertension in female intrauterine growth–restricted offspring (i.e., low birth weight) rats that occurs with advanced age (160). In addition to the effects of GPER agonism via G-1, G36 prevented AngII-induced hypertension in mice through a unique mechanism resulting from the downregulation of Nox1 with the subsequent lack of reactive oxygen species production involved in AngII-induced vasoconstriction and thus hypertension (161). In a rat model of diabetic cardiomyopathy, mean arterial pressure, cardiac weight, and atherogenic and cardiovascular risk indices were improved by E2 and G-1 treatment, with the salutary effects of E2 inhibited by G15 (162). In addition to arterial hypertension, G-1 was effective in treating pulmonary arterial hypertension, reversing both cardiac and skeletal muscle functional aberrations in ovariectomized female (163) and male (164) mice.

Atherosclerosis, which can lead to coronary artery disease, results from elevated lipid levels in the blood and a chronic inflammatory state. G-1 protected against the development of atherosclerosis through multiple actions in both diet-induced (165) and genetic models (166). First, G-1 reduced plasma cholesterol levels (see below) (144). Second, G-1 induced differentiation and inhibited coronary smooth muscle cell proliferation (167). Third, G-1 reduced inflammation in a diet-induced model of atherosclerosis in mice (165). Consistent with an anti-inflammatory role for GPER, GPER KO mice exhibited increased accumulation of inflammatory cells as well as atherosclerosis in both ovary-intact and ovariectomized mice (165). Fourth, G-1, as well as E2, induces nitric oxide production in human endothelial cells (both inhibited by G36) (165) and enhanced vasodilation (166). Endothelial dysfunction and reduced NO production are hallmarks of atherosclerosis and vascular disease (151, 168).

### Endocrinology and Metabolism

Metabolic homeostasis is differentially regulated in males and females (169, 170), with premenopausal women exhibiting a lower incidence of obesity and diabetes compared to age-matched men. These protective effects, presumably a result of estrogen, are lost following menopause (171, 172). This sex difference, as well as the effects of estrogen deprivation, is also present in mice (173, 174). Estrogen replacement therapy in postmenopausal women, as well as in ovariectomized mice, can alleviate weight gain and its associated adverse metabolic effects (173–176).

GPER expression is associated with body weight, energy expenditure, and glucose homeostasis. This is evidenced by the fact that GPER KO mice exhibited increased body weight and adiposity (in both the visceral and subcutaneous depots), dyslipidemia, and insulin resistance and glucose intolerance (153, 177–180). That GPER modulates basal metabolism was concluded from the fact that no changes in either steady-state daily food intake or locomotor activity were observed, but energy expenditure decreased in GPER KO mice, consistent with the observation of decreased brown adipose tissue expression of the thermogenic genes uncoupling protein 1 and β_3_-adrenergic receptor (177, 179). Interestingly, although there was no overall difference in food intake, female GPER KO mice exhibited a lower sensitivity to the short-term feeding inhibition of leptin and cholecystokinin (179). Consistent with this effect, G-1 treatment of ovariectomized rats led to an acute transient decrease in food intake (181). Employing models of obesity through either estrogen deprivation (i.e., ovariectomy) or a high-fat diet (HFD), chronic G-1 treatment following weight gain led to weight and adipose tissue loss, improved levels of circulating lipids, and increased energy expenditure with no changes in food consumption or locomotion (144). Similarly, no changes in either lean mass or bone density/mineral content were observed. In both white and brown adipose tissue, as well as skeletal muscle, G-1 treatment increased the expression of genes involved in mitochondrial biogenesis and fatty acid oxidation while reducing expression of many genes involved in inflammation, hypoxia, and angiogenesis (144). Importantly, as previously observed (165), G-1 treatment of ovariectomized mice did not lead to uterine imbibition (144), as occurs with estrogen supplementation (182).

GPER KO mice also exhibited higher plasma glucose and impaired insulin sensitivity and glucose tolerance as well as defective glucose- and estrogen-stimulated insulin secretion (177–179). In a streptozotocin-induced model of type 1 diabetes, female GPER KO mice displayed decreased pancreatic insulin and pancreatic β cell content as well as higher blood glucose (183). In addition to promoting islet survival (183), GPER mediated insulin secretion in isolated islets in response to E2 and G-1, both of which were reduced by GPER inhibition with G15 or in islets from GPER KO mice (184). Lastly, ovariectomized wild-type but not GPER KO mice responded to acute and chronic estrogen treatment with improved glucose homeostasis, further revealing the role of GPER in estrogen function in vivo (178, 179). The models described above also resulted in metabolic dysfunction, including insulin resistance and glucose intolerance. Treatment with G-1 also led to improvements in glucose homeostasis, as revealed by glucose- and insulin-tolerance tests and reduced fasting glucose and insulin concentrations (144). Employing ovariectomy, streptozotocin, and a HFD to create a rat model of severe postmenopausal type 2 diabetes, one study found that estrogen and G-1 treatment improved fasting blood glucose and HOMA-IR (homeo-static model assessment for insulin resistance), with estrogen’s salutary effects reversed by G15 (162). That GPER also functions to enhance insulin secretion in humans has been demonstrated in pancreatic islets isolated from type 2 diabetic patients where glucose-stimulated insulin secretion increased, while glucagon and somatostatin secretion decreased, upon G-1 stimulation (185, 186).

### Actions of GPER-Selective Ligands in Other Systems

GPER is expressed in and plays multiple roles in the skin. GPER’s role in estrogen-induced melanogenesis suggests GPER modulators could find applications in chloasma and other skin pigmentation disorders (187, 188). In skin and soft tissue infection resulting from *Staphylococcus aureus*, G-1 decreased dermonecrosis, likely through reduced overall neutrophil accumulation, and increased bacterial clearance in the absence of direct bactericidal effects (189). The observed sex difference and role of estrogen in wound healing (190, 191), along with reduced healing in GPER KO mice (R. Ko, O. Davidson, K. Ahmed, R. Clark, J. Brandenburg, et al., unpublished results), suggest additional opportunities for GPER agonist therapy in skin conditions and wound healing.

With respect to the hepatobiliary system, estrogen has multiple functions in both the liver and gall bladder, protecting liver function and reducing steatohepatitis, while promoting gallstone formation. Estrogen and genistein, in part through GPER, protected hepatocytes from mitochondrial dysfunction and triglyceride accumulation (192). DHEA, through conversion to estrogen, which then acts through GPER, reduced murine nonalcoholic steatohepatitis (47). Estrogen-promoted formation of gallstones involves both GPER and ERα, with distinct cholesterol crystallization pathways for the two receptors described (193). Furthermore, in GPER KO mice, gallstone formation was absent, whereas it was increased by treatment of wild-type mice with estrogen and G-1 (193, 194). Conversely, gallstone formation was reduced by selective GPER antagonists such as the G36 analog, CIMBA, suggesting that targeting GPER with antagonists may represent a therapeutic opportunity for this condition (110).

In the gastrointestinal tract, G-1 reduced motor function (i.e., muscle contractility and therefore motility), visceral pain (195, 196), and reperfusion injury following intestinal ischemia/reperfusion through decreased colonic crypt cell injury (197). G-1 also reduced mortality and tissue damage in a model of Crohn’s disease (198), and GPER activation also attenuated intestinal inflammation in a model of acute colitis, resulting in improved intestinal mucosal barrier function (199, 200). Intestinal expression of GPER appears to be increased in Crohn’s disease (198), ulcerative colitis (201), and irritable bowel syndrome (202).

GPER regulates multiple aspects of kidney function, including renal artery and interlobular artery vascular tone (203, 204). Estrogen and G-1, through GPER activation, stimulated H^+^-ATPase activity in renal tubular intercalated cells (205) and regulated Na^+^ excretion in vivo (206). Icariin, a GPER agonist, protected kidney podocytes from apoptosis (207), and in hypertensive nephropathy, G-1 reduced proteinuria, without accompanying changes in blood pressure (208, 209). G-1 also reduced renal cell injury resulting from methotrexate treatment (210). Interestingly, GPER KO mice exhibited greatly reduced age-associated renal fibrosis and kidney disease, likely through the regulation of Nox1, as observed in the heart and vasculature, suggesting a therapeutic role for GPER antagonists in chronic kidney disease (161, 211).

Many salutary effects of estrogen in nonreproductive tissues and diseases appear to involve anti-inflammatory effects that are at least in part mediated through GPER, which is broadly expressed in immune cells (145). Consistent with this, GPER KO mice exhibited increased inflammation in many models (135, 165, 177, 179, 212), whereas G-1 administration reduced inflammation in multiple murine models, including lung allergy with airway hyperresponsiveness (213), chronic obesity and diabetes (144), inflammatory bowel diseases (198, 214), and chronic neurological diseases (212, 215–218). Among its actions, G-1 promoted production of the anti-inflammatory cytokine IL-10 in Th17 cells (219, 220) and reduced the production of lipopolysaccharide-induced cytokines in macrophages (217). GPER activity was also sufficient to protect fetal development and neonatal viability in times of maternal infection and inflammation of the placenta, suggesting a therapeutic avenue through G-1 (221).

GPER plays extensive roles in the central and peripheral nervous system, as demonstrated by the array of protective G-1 actions in acute and chronic neurological/neurodegenerative diseases (218). In a multiple sclerosis model (experimental autoimmune encephalomyelitis), G-1 both reduced the severity and delayed the onset of symptoms through reductions in immune reactivity (212, 217). In models of Alzheimer’s and Parkinson’s disease and after traumatic brain injury, G-1 improved multiple measures of neurological functions in part through reducing neuroinflammation (222–225). In traumatic brain and spinal cord injury models, G-1 provided protection (223, 226, 227). In stroke models, G-1 reduced infarct size, blood-brain barrier permeability, and stroke-induced immunosuppression (228–232) through improved neuronal survival signaling (228, 233) and improved cerebral microvascular function (231), restoring autophagy in astrocytes (234) or inhibiting TL4-mediated microglial inflammation (229). GPER activation with G-1 also exhibited antidepressant and anxiolytic effects (81, 235), supported by studies in GPER KO rats (236). Cognition, learning, memory, and other behavioral effects (e.g., lordosis) are also associated with GPER via the actions of G-1 (222, 224, 237–241).

## CONCLUSIONS AND FUTURE DIRECTIONS

Since our last review on the pharmacology of GPER in 2015 (7), significant advances have been made in understanding the functions of GPER and the potential applications of GPER-targeted ligands (both agonists and antagonists). New natural and synthetic compounds have been identified as GPER agonists and antagonists, suggesting roles for GPER in the beneficial effects of phytoestrogens as well as the harmful effects of endocrine disruptors. The continuing identification of novel GPER actions and applications, frequently demonstrated through the use of GPER-targeted ligands, in virtually every system of the body, portends opportunities for therapeutic development of such targeted ligands and the necessity of assessing GPER effects of current and developing drugs. With the advancement of G-1 to Phase I/II clinical trials in 2020 for advanced cancers (https://clinicaltrials.gov/ct2/show/NCT04130516), and intriguing opportunities in metabolic disorders and cardiovascular, kidney, and hepatobiliary diseases, not to mention immune, neurological, gastrointestinal, and infectious diseases, GPER-targeted compounds may find broad use in the pharmacopoeia.
